# Tibial torsion in non-arthritic Indian adults: A computer tomography study of 100 limbs

**DOI:** 10.4103/0019-5413.41854

**Published:** 2008

**Authors:** Arun B Mullaji, Amit K Sharma, Satyajit V Marawar, AF Kohli

**Affiliations:** Department of Orthopedic Surgery, Breach Candy Hospital, Mumbai, India; 1 Department of Orthopedic Surgery, KEM Hospital, Mumbai, India

**Keywords:** Computer tomography, Indian, non-arthritic limbs, tibial torsion

## Abstract

**Background:**

Knowledge of normal tibial torsion is mandatory during total knee replacement (TKR), deformity correction and fracture management of tibia. Different values of tibial torsion have been found in different races due to biological and mechanical factors. Value of normal tibial torsion in Indian limbs is not known, hence this study to determine the norm of tibial torsional value in normal Indian population.

**Materials and Methods:**

Computer tomography (CT) scans were performed in 100 non-arthritic limbs of 50 Indian adults (42 males, eight females; age 26-40 years). Value of tibial torsion was measured using dorsal tangent to tibial condyles proximally and bimalleolar axis distally.

**Results:**

Normal tibial torsion was found to be 21.6 ± 7.6 (range 4.8 to 39.5) with none of the values in internal rotation. Right tibia was externally rotated by 2 degrees as compared to the left side (*P* 0.029). No significant difference was found in male and female subjects. Value of tibial torsion was less than in Caucasian limbs, but was comparable to Japanese limbs when studies using similar measurement technique were compared.

**Conclusions:**

Indian limbs have less tibial torsion than Caucasian limbs but the value of tibial torsion is comparable to Japanese limbs.

## INTRODUCTION

Tibial torsion is defined as torsion of tibia along its longitudinal axis. Deformity of the lower limb in the coronal plane has been widely investigated and found to be associated with the development of osteoarthritis. However, rotational deformities of the lower limb are still a controversial subject. Some surgeons have advocated the correction of rotational deformity during total knee replacement (TKR) and during treatment of complex tibial fractures.^1^–^3^ However, torsion of normal tibia should be known to achieve the right amount of rotation,^4^ specially when both the lower limbs are affected and normal reference value from the opposite side is not available.^2^,^5^–^8^

Position and other attitude of posture will produce rotational changes in the tibiae, depending upon the direction of the rotary stress applied. Racial and geographical variations have been found in the tibial torsion due to different ways of sitting and positional pressures on the leg.^9^–^16^ For correction of rotational deformity of the limb, norms for that particular race are required. No study to determine normal tibial torsion has been performed in the Indian population.

Various clinical,^2^,^6^,^11^,^16^–^21^ fluoroscopy,^22^–^24^ ultrasonography (USG)^4^,^10^,^25^ and magnetic resonance imaging (MRI)-based methods^7^ have been proposed in the literature for tibial torsion measurement. We used computer tomography (CT) scan for tibial torsion measurements in our study as CT is indisputably the best imaging modality for bone and identification of the reference points is quite easy.^3^,^26^,^27^

The aim of the present study was to determine norms of tibial torsional value in the normal Indian population and to find the difference between Japanese, Caucasian and Indian tibial torsion, if any, measured by similar method.

## MATERIALS AND METHODS

One hundred limbs were studied in 50 subjects (42 males, eight females). All the patients were 26-40 (mean-31.3 ± 2.9) years of age. All subjects were undergoing CT study for non-orthopedic illness with no lower limb symptoms. Relevant history was taken and physical examination conducted on every subject. The subjects having knee pain, previous trauma or surgery to leg, excessive ligamentous laxity, and deformity of the limb were excluded from the study. Written permission of the subjects and local ethics committee approval was taken for the study.

A long leg radiograph of the subjects on a long leg plate was taken in the standing position to ensure absence of knee arthritis and limb deformity. Femoro-tibial angle was measured on this radiograph as an angle between the mechanical axis of the femur and tibia. A CT study of the lower limbs was performed. Subjects laid down supine on the CT table with their lower limbs parallel to each other. Limbs were kept in neutral position by keeping the patella facing towards the roof. Limbs were immobilized in this position during the scanning procedure with the help of a plastic derotation boot with attached bar. The CT cuts were taken in 1 mm thick slices from the superior pole of the patella to 1 cm below the tibial tuberosity. Additional CT cuts were taken from 3 cm above the ankle joint to the tip of the fibula (lateral malleolus). Special care was taken to avoid any movement of the limb during the entire procedure. Simultaneous CT cuts of both the limbs were taken.

**Figure 1:**
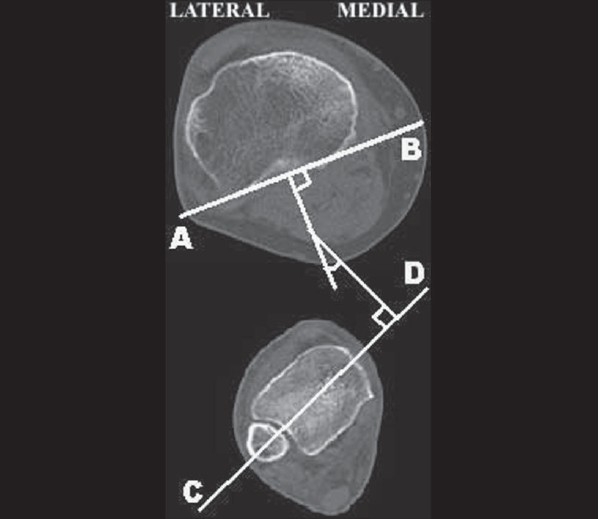
Tibial torsion:- Measurement on computer tomography scan line AB - Proximal reference line (Posterior condylar axis), line CD - Distal reference (Bimalleolar axis); Angle between line AB and CD is tibial torsion

A tangent to the most posterior prominent point of the medial and lateral tibial condyles was drawn on CT cuts of the upper end of the tibia. This was used as proximal reference line. Distally, a line was drawn across the midpoints of the medial and lateral malleolus at the level of the ankle joint. Lateral angle between these two lines was recorded as tibial torsion [Figure 1]. Values from the right and left leg of all the subjects were measured.

All the values were entered in Microsoft Excel^®^ spreadsheet (Microsoft Corporation, Redmond, WA, USA) on a predefined protocol. Mean, standard deviation and range of the values were obtained. Student's t test was used to assess significance of difference between the groups at a *P* value of 0.05.

## RESULTS

**Figure 2:**
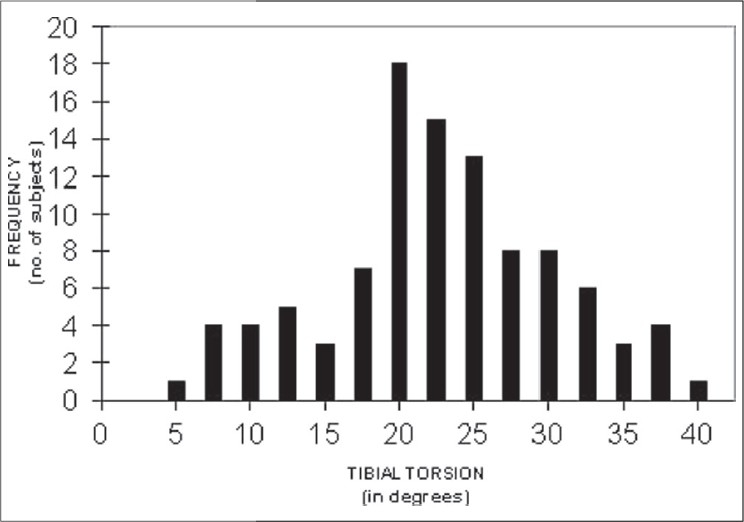
Frequency distribution curve - Tibial torsion

Mean tibial torsion in 100 legs was 21.6 ± 7.6 degrees (range-4.8-39.5) [Figure 2]. None of the tibia was in internal rotation. Difference between right (22.6 ± 7.8 degrees) and left (20.6 ± 7.4 degrees) side was significant (*P*value 0.029). Though male limbs had higher tibial torsion (22.2 ± 7.6 degrees, n-84) than female limbs (18.4 ± 7.5 degrees, n-16), this difference was not found to be statistically significant (*P* 0.07). Mean femoro-tibial angle was 179.6 ± 2.1 degrees (176.0-185.0) with no difference in the value of male and female subjects (*P* 0.7). Similarly, there was no right-left difference in femoro-tibial alignment (*P*0.5). Tibial torsion in 10 limbs was measured thrice by a single observer at three different occasions at one-week interval between two measurement sessions and by three different observers individually. Inter-observer and intra-observer variation was found to be 0.7 ± 1.3 and 1.1 ± 1.7, respectively. Kappa value for the inter-observer reliability and intra-observer reliability was 0.84 and 0.89, respectively.

## DISCUSSION

Abnormal rotation of the lower limb may produce excessive stress on the implant in a post-TKR patient and hence rotational alignment of the lower limb needs to be addressed during TKR.^4^ Rotational deformity may lead to early onset osteoarthritis.^1^,^5^,^10^,^13^,^21^,^28^–^31^ Tibial torsion plays an important role in patello-femoral arthritis,^32^ genu valgum and varum.^18^ Torsional deformity of the tibia has been found to be associated with club feet, cerebral palsy and other neuromuscular problems.^25^,^33^

There is no consensus in the literature regarding proximal and distal reference points to measure tibial torsion.^9^ Various proximal and distal reference points have been proposed in different studies to measure tibial torsion and hence, great variation has arisen in the values obtained between different studies.^9^,^17^ Posterior surface of femoral condyles^14^,^22^ does not give true tibial torsional values as knee joint rotation will affect the values measured. It is more representative of leg torsion than tibial torsion. It is difficult to define the transtibial axis^9^,^19^,^26^,^29^,^31^,^34^ which is the line joining the most prominent medial and lateral points of the tibial plateau because of the rounded shape of the tibial articular surface. However, a line tangent to the posterior surface of the medial and lateral tibial condyles can always be defined.^1^,^4^,^6^,^9^,^25^,^27^,^29^ Eckhoff *et al.*, in their three-dimensional CT study proved that the posterior condylar axis is a reliable reference axis and the effect of some amount of varus-valgus slope of CT cuts is negligible on the measured values.^9^ Distal reference points in the literature are also as varied as proximal reference points. Two different terms are in vogue; tibial torsion and tibio-fibular torsion. Tibial torsion is the term used when the distal axis is drawn with the help of reference points on the tibia only.^1^,^4^,^7^,^25^ In contrast, in tibio-fibular torsion, both tibia and fibula are used to draw distal axis.^18^,^20^,^26^,^30^ Rosen *et al.*,^18^ gave the concept of tibio-fibular torsion rather than tibial torsion. Knee and ankle joints are hinge joints and any deviation of their movement axis from normal will produce deleterious effects on these joints if the resultant axes of movement of these joints are not parallel.^18^,^29^ Alignment of the ankle joint is determined by both the tibia and the fibula. Hence, it is more practical to measure tibial torsion by taking both bones into consideration rather than the tibia alone and thus bimalleolar axis is more useful to determine tibial torsion (or tibio-fibular torsion). Different methods have been used to draw bimalleolar axis.^1^,^7^,^26^ However, a line joining the center of both the malleoli is most widely used and has been found to be an easy and reliable method to draw distal tibial axis.^8^,^9^,^12^,^14^,^17^,^19^,^21^,^26^,^27^,^30^,^31^,^34^ In the present study, we used a line tangent to the posterior condylar surface of the tibia as proximal reference, and bimalleolar axis as distal reference axis, and the term tibial torsion, rather than tibio-fibular torsion, has been used in this study.

Ethnic and gender differences have been found in the value of tibial torsion.^9^–^16^ It has been found that different lifestyles and postures adopted among different ethnic groups lead to differences in rotation of the tibia between them. Japanese people sit in the position of “Seiza”, a position in which they sit on the ground with their legs folded underneath and feet turned inward with the buttocks resting directly on the feet. This position is assumed to exert an internal rotation force on the tibia leading to increased internal rotation of the tibia, as has been found in various studies.^11^,^16^ Indian subjects sit on the ground in cross-legged position. Sitting cross-legged in Indian fashion has been proposed as a method of treatment for pediatric tibial intorsion assuming that this position will force external rotation of the tibia.^35^

Tibial torsion found in our study was 21.6 ± 7.6 degrees, which is markedly less than the values obtained from Caucasian limbs (Table 1).^8^,^14^,^17^,^29^,^30^ However, in most of these studies, subjects were of the older age group with wide age range as compared to our study in which mean age of the subjects were 31.3 ± 2.9 years with a range of 26-40 years. Though, subjects chosen in other studies were claimed to be having normal limbs without any evidence of arthritis or coronal plane deformity, change in the tibial torsion with advance in the age of the studied subjects cannot be ruled out.^9^ In contrast to that, tibial torsion of Japanese subjects was quite close to our value.^31^ Whether this reflects any similarity in the biological or postural behavior of Japanese and Indians is not known and requires further study. We studied tibial torsion in subjects 26-40 years of age to avoid the possible effect of growing bones of the younger age group or arthritis of knee in the older age group on tibial torsion.

**Table 1:** Comparative analysis of Tibial torsion in various studies using dorsal tangent to proximal tibial condyles and bimalleolar axis*

Study group	Tibial torsion (in degrees)	Right (RT) versus left (LT) (in degrees)	Male versus female (in degrees)	Age (in years)
Present Study	21.6 ± 7.6	RT- 22.6 ± 7.8	Male- 22.2 ± 7.6	31.3 ± 2.9
	(4.8-39.5)	LT- 20.6 ± 7.4	Female- 18.4 ± 7.5	(26-40)
Laasonen *et al.*^29^	31.3 (6.7-48.3)	—	—	(49-72)
Lang *et al.*^17^	33.7 ± 10.0	RT-34.4 ± 8.1	—	(26-73)
		LT-33.1 ± 12.2	—	
Reikeras *et al.*^14^	—	—	Female-RT 32.3 ± 8.5	Males-35
			LT 30.7 ± 10.4	Females-39
			Male-RT 35.3 ± 7.6	(16-70)
			LT 34.0 ± 10.3	
Sayli *et al.*^30^	—	RT-31.07	Female-RT 29.7	Median age 31.4
		LT-30.02	LT 25.9	(15-76)
			Male- RT 32.7	
			LT 35.2	
Strecker *et al.*^8^	34.8 ± 15.8	RT-36.4	—	Male-32.3
		LT-33.0		(18-78)
				Females-35.8
				(16-73)
Yagi *et al.*^31^	23.5 (14-33)	—	—	64 (42-82)

* All values are in mean ± standard deviation (range)

In a clinical study by Tamari *et al.*,^15^ it was found that Japanese subjects had more varus than Australian Caucasians. Tibiofibular torsion was found to be significantly more in younger and middle age group females than their older counterparts, but similar difference was not found in male subjects. An ethnic difference was not found between Japanese and Australian Caucasians when their tibio-fibular torsion was compared, though ethnic and gender differences were found in femoral antetorsion and femoro-tibial angle. However, they used clinical method to measure tibial torsion.

No right to left difference between values of tibial torsion have been found in many studies.^14^,^17^,^19^,^26^,^34^ However, we found a statistically significant difference in the right and left tibial torsion as was found in some other studies also.^4^,^8^,^11^,^22^,^23^ Right tibial torsion was always greater than the left. Clementz *et al.*,^22^,^23^ found a right to left difference ranging from -11 to 15 degrees with a mean difference of 2.5 degrees higher on the right side. In 25% of the subjects, this difference was at least 6 degrees. Mean right-left difference was 2.0 degrees in our study. It has been postulated that limbs tend to rotate more on the right side than left, and hence, right tibia go more into external torsion and left in internal rotation.^34^ However, difference in the values were not great and the clinical significance of this right-left difference is not known.

The results of our study should be interpreted with caution as measured value changes with change in the proximal and distal reference points, age range, ethnic and gender characteristics of the study group and method of the measurements.^9^ Causes of reduced external torsion in Indian tibia require further study. Tibial torsional values from our study should help the surgeon to determine the normal range of tibial torsion to be achieved during TKR, management of complex deformity of the tibia or fracture treatment, especially when the deformity is bilateral.

## CONCLUSION

The value of tibial torsion in our study was 21.6 ± 7.6 degrees in 26-40 years age group. We found tibial torsion on right side greater than left by 2 degree.
